# Supplementary material for: Splice-Junction-Based Mapping of Alternative Isoforms in the Human Proteome
Source: Cell Rep. Author manuscript; Available in PMC 2020 Jan 15. (PMC6961840; doi:10.1016/j.celrep.2019.11.026)
Supplement: 2 [file NIHMS1546469-supplement-2.zip › DF1/PXD010154/Liver/Liver_65_BHMT2_AGSNVMQTFTFSASEDNMESKGLPS.pdf]

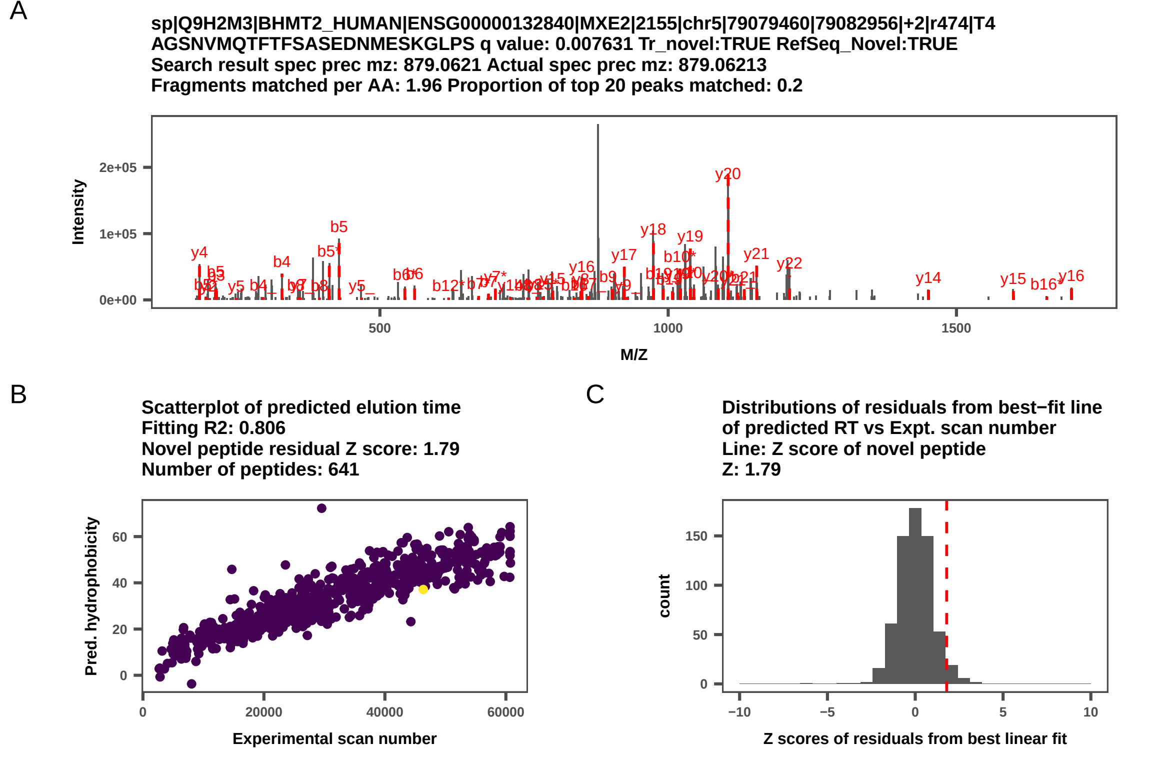
A
sp|Q9H2M3|BHMT2_HUMAN|ENSG00000132840|MXE2|2155|chr5|79079460|79082956|+2|r474|T4
AGSNVMQTFTFSASEDNMESKGLPS q value: 0.007631 Tr_novel:TRUE RefSeq_Novel:TRUE
Search result spec prec mz: 879.0621 Actual spec prec mz: 879.06213
Fragments matched per AA: 1.96 Proportion of top 20 peaks matched: 0.2
Intensity
2e+05
1e+05
0e+00
y20
b5
y18
y19
y4
b5*
y17
b10*
y21
b4
y22
y16
y14
y15
b16*
y16
500
1000
1500
M/Z
B
Scatterplot of predicted elution time
Fitting R2: 0.806
Novel peptide residual Z score: 1.79
Number of peptides: 641
Pred. hydrophobicity
60
40
20
0
0
20000
40000
60000
Experimental scan number
C
Distributions of residuals from best−fit line
of predicted RT vs Expt. scan number
Line: Z score of novel peptide
Z: 1.79
count
150
100
50
0
−10
−5
0
5
10
Z scores of residuals from best linear fit
